# Uncertainty analysis of vegetation distribution in the northern high latitudes during the 21st century with a dynamic vegetation model

**DOI:** 10.1002/ece3.85

**Published:** 2012-03

**Authors:** Yueyang Jiang, Qianlai Zhuang, Sibyll Schaphoff, Stephen Sitch, Andrei Sokolov, David Kicklighter, Jerry Melillo

**Affiliations:** 1 Department of Earth and Atmospheric Sciences, Purdue University,West Lafayette, Indiana; 2 Department of Earth and Atmospheric Sciences and Department of Agronomy, Purdue University,West Lafayette, Indiana; 3 Potsdam Institute for Climate Impact Research,P.O. Box 601203, D-14412 Potsdam, Germany; 4 Met Office Hadley Centre, JCHMR, Maclean Building,Wallingford OX10 8BB, United Kingdom; 5 Department of Earth, Atmospheric, and Planetary Sciences, Massachusetts Institute of Technology,Cambridge, Massachusetts; 6 The Ecosystems Center, Marine Biological Laboratory at Woods Hole,Massachusetts

**Keywords:** Climate-induced uncertainty, greenness migration, prameter importance, parameter-induced uncertainty, sensitivity analysis, vegetation redistribution

## Abstract

This study aims to assess how high-latitude vegetation may respond under various climate scenarios during the 21st century with a focus on analyzing model parameters induced uncertainty and how this uncertainty compares to the uncertainty induced by various climates. The analysis was based on a set of 10,000 Monte Carlo ensemble Lund-Potsdam-Jena (LPJ) simulations for the northern high latitudes (45^o^N and polewards) for the period 1900–2100. The LPJ Dynamic Global Vegetation Model (LPJ-DGVM) was run under contemporary and future climates from four Special Report Emission Scenarios (SRES), A1FI, A2, B1, and B2, based on the Hadley Centre General Circulation Model (GCM), and six climate scenarios, X901M, X902L, X903H, X904M, X905L, and X906H from the Integrated Global System Model (IGSM) at the Massachusetts Institute of Technology (MIT). In the current dynamic vegetation model, some parameters are more important than others in determining the vegetation distribution. Parameters that control plant carbon uptake and light-use efficiency have the predominant influence on the vegetation distribution of both woody and herbaceous plant functional types. The relative importance of different parameters varies temporally and spatially and is influenced by climate inputs. In addition to climate, these parameters play an important role in determining the vegetation distribution in the region. The parameter-based uncertainties contribute most to the total uncertainty. The current warming conditions lead to a complexity of vegetation responses in the region. Temperate trees will be more sensitive to climate variability, compared with boreal forest trees and C3 perennial grasses. This sensitivity would result in a unanimous northward greenness migration due to anomalous warming in the northern high latitudes. Temporally, boreal needleleaved evergreen plants are projected to decline considerably, and a large portion of C3 perennial grass is projected to disappear by the end of the 21st century. In contrast, the area of temperate trees would increase, especially under the most extreme A1FI scenario. As the warming continues, the northward greenness expansion in the Arctic region could continue.

## Introduction

Vegetation distribution plays a critical role in atmospheric dynamics through its effects on the earth's surface and on atmospheric water cycling (D’Odorico et al. 2006; Saco et al. 2007; Thompson et al. 2010), carbon cycling (Lucht et al. 2006; Bounoua et al. 2010), and the surface energy budget (Kim and Wang 2005). In the northern high latitudes, the large-scale vegetation distribution is significantly controlled by climate (Woodward 1987), which has had a clear warming trend for the past three decades (ACIA 2005; Chapman and Walsh 2007). This trend is expected to continue through the 21st century (Overland et al. 2004; Wolf et al. 2008).

Vegetation has been extensively observed to have a considerable response to this warming trend (Tape et al. 2006; Soja et al. 2007), and this response could be potentially stronger in the next hundred years (Gerber et al. 2004; Lucht et al. 2006; Walker et al. 2006). For instance, several studies have shown a clear warming temperature trend caused northward and upslope migration of treelines and an alteration in the current mosaic structure of boreal forests in the pan-Arctic region (Soja et al. 2007; Shuman and Shugart 2009). Some other studies presented an increase in the abundance and extent of shrubs responding to the climate warming in the Arctic region over the past several decades (Tape et al. 2006). Studies such as Tchebakova et al. (2009) predicted that the drier climate would cause the Sibrean forest to decrease and shift northward. By the end of the 21^st^ century, forest-steppe and steppe ecosystems were predicted to dominate more than half of the Siberian zone (Tchebakova et al. 2009).

The shift in vegetation distribution could alter the ecosystem energy balance and carbon cycle, further affecting the climate system (Chapin III et al. 2000; Beringer et al. 2005). For instance, Thompson et al. (2004) showed a dramatic decrease in albedo along the vegetation gradient from tundra to forest due to increasing canopy complexity. Consequently, this decrease in albedo could exert a positive feedback on radiative forcing and amplify atmospheric warming (Euskirchen et al. 2009). In addition, Bonan et al. (2003) has shown that the northward expansion of boreal forest into tundra could remove carbon from the atmosphere, thereby resulting in a climate cooling effect that compensates for the warming effect due to reduced surface albedo (Betts 2000). Many other studies (Wilmking et al. 2004; Walker et al. 2006; Euskirchen et al. 2009) also emphasize the complexity of plant feedbacks to climate change, however the uncertainty is considerably high (Cox et al. 2004; Schaphoff et al. 2006).

Assessing potential changes in vegetation distribution in boreal ecosystems is essential for understanding the environmental response to changing climate (Cramer et al. 2001). To date, a number of dynamic global vegetation models (DGVMs) are promising to project future changes in vegetation distribution, including its future structure and composition (Cramer et al. 2001; Sitch et al. 2003). However, uncertainties in model parameter values and uncertainties in the climate inputs lead to considerably uncertain results from these projections. Previous sensitivity and uncertainty studies have mostly focused on carbon and water balances (Knorr et al. 2005; Shuman and Shugart 2009; Tang and Zhuang 2009). Zaehle et al. (2005) has already examined the effects of parameter uncertainty on contemporary distributions of plant functional types (PFTs) estimated by the Lund-Potsdam-Jena DGVM (LPJ-DGVM; Sitch et al. 2003; Gerten et al. 2004). However, that study only reported the effects of climate change on the future distributions of tropical and temperate PFTs, based on only a single climate projection (IS92a HadCM2-SUL, Mitchell et al. 1995;Johns et al. 1997). This study aims to assess how high-latitude vegetation may respond under various input emission and climate scenarios over the 21st century, with a focus on how changes in model parameters may influence the model output, and how this uncertainty then compares to uncertainty generated from various climate inputs.

In this study, we built upon the Zaehle et al. (2005) study and quantified the uncertainty of the distribution of six PFTs found in the northern high latitudes (above 45^o^N) for several emission and climate scenarios using the LPJ-DGVM. These PFTs include temperate needleleaved evergreen (TNE) woody, temperate broadleaved evergreen (TBE) woody, temperate broadleaved summergreen (TBS) woody, boreal needleleaved evergreen (BNE) woody, boreal summergreen woody (BSW), and C3 perennial grass (CPG). We further examined how the interactions between climate inputs and parameterization affect the uncertainty of vegetation distributions (Overland et al. 2004; Weng and Zhou 2006; Tchebakova et al. 2009). By helping to better understand these interactions, this study represents a step toward better projection of vegetation distribution in the northern high latitudes. In addition, the quantification of probabilities of future vegetation distribution will be useful for ecosystem management and climate policy-making.

## Method

### Overview

Ten sets of climate change scenarios (see section Forcing Data) were used to drive LPJ-DGVM simulations in the northern high latitudes from 2001 to 2100. We first used a Latin Hypercube sampler (LHS) algorithm (Iman and Helton 1988) to develop 1000 sets of parameter values. We then conducted Monte Carlo (MC) simulations with LPJ-DGVM using each of the 1000 sets of parameter values under each of the 10 climate scenarios, for a total of 10,000 simulations. Based on these MC ensemble simulations, we quantitatively ranked parameter importance using the Partial Rank Correlation Coefficient (PRCC; Saltelli 2002; Marino et al. 2008), and described the spatial characteristics of their influences. We further examined the changes in the coverage of six PFTs during the 21st century under these climate scenarios. Finally, the sensitivity and uncertainty of changes in vegetation distributions to climate inputs and parameters were analyzed.

### Description of LPJ-DGVM model

The LPJ-DGVM (Sitch et al. 2003; Gerten et al. 2004) is a process-based model that explicitly combines biogeography and biogeochemistry modeling approaches in a modular framework to simulate the spatio-temporal dynamics of terrestrial vegetation and land–atmosphere carbon and water exchanges. The LPJ-DGVM is driven by annual atmospheric CO_2_ concentration and soil type, together with monthly climate data on air temperature, precipitation, and fractional cloud cover. It simulates photosynthesis, plant distribution, and competition for water and light of nine PFTs, which are defined using physiological parameters influencing growth. The establishment of plants is determined by bioclimatic limits. Mortality is governed by the interactions among light competition, low growth efficiency, heat mortality, and when bioclimatic limits are exceeded for an extended period.

In a single grid cell, each PFT is represented in terms of the overall foliage projective cover (FPC), which is determined by the product of individual FPC with crown area and population density (eq. 8 in Sitch et al. 2003). The individual FPC is calculated by the Lambert–Beer law (Monsi and Saeki 1953; Specht 1981). The sum of FPCs through all PFTs is between 0 and 1, with the difference between 1 and the sum of FPC for all PFTs representing the bare ground, or in other words, the area remaining to be colonized. Resource competition and differential responses to fire between PFTs influence their relative annual fractional cover and their population density. An individual PFT can migrate into new regions if its bioclimatic limits and competition with other PFTs allow establishment and successful plant growth (Gerber et al. 2004).

There are several rules and constraints for the calculation of FPC in a grid cell, according to Sitch et al. (2003). For instance, establishment takes place in each simulation year. The annual establishment of new individuals depends on the potential establishment rate (*est*_max_), and on the current fraction of the grid cell devoid of woody plants. The annual establishment of new individuals also declines in proportion with canopy light attenuation when the sum of woody FPCs exceeds 0.95 and approaches 1, simulating a decline in establishment success with canopy closure (Prentice et al. 1992). The net effect of establishment is always a marginal increase in FPC. Another constraint for the calculation of FPC in a grid cell is that if the sum of woody FPCs is greater than unity in a particular year, the herbaceous biomass is first reduced, representing the competitive dominance of the taller-growing woody PFTs. In addition, heat stress, meaning depressed growth efficiency or a situation in which a PFT's bioclimatic limits are exceeded, could lead to additional mortality. The net effect of mortality is a marginal decrease in FPC, creating new space for PFT expansion by growth and establishment (Smith et al. 2001). More details about the LPJ model are presented in Sitch et al. (2003) and Gerten et al. (2004).

In LPJ, the actual intercellular-to-atmospheric CO_2_ concentration ratio is simulated using a coupled photosynthesis and water balance canopy conductance scheme (Sitch et al. 2003). The elevated CO_2_ concentration could enhance the plant productivity through two mechanisms. First, the increased intercellular partial pressure of CO_2_ directly increases the assimilation of C3 plants, and inhibits photorespiration, thereby leading to more efficient photosynthesis. Second, CO_2_ fertilization could lower stomatal conductance and reduce water loss during transpiration, thereby leading to a higher water use efficiency, which could further lengthen the growing season and increase net primary production (Prentice et al. 2001) and potentially enable the expansion of PFTs, even under drought conditions (Neilson and Drapek 1998). The LPJ-DGVM has been proven to reasonably assess vegetation redistribution due to environmental changes at large scales (Sitch et al. 2003; Schaphoff et al. 2006). Zaehle et al. (2005) has shown that there are a number of key parameters in the LPJ-DGVM that have strong effects on the establishment, growth, and mortality of PFTs. The values of these parameters, which are stratified by PFT, are normally determined through model calibration or empirical studies (Prentice et al. 1992; Smith et al. 2001).

### Forcing data

A set of 0.5^o^× 0.5^o^ gridded monthly climate data (air temperature, precipitation, and cloudiness), obtained from the Climate Research Unit (CRU) of the University of East Anglia (New et al. 2002; Mitchell and Jones 2005), were used to drive each simulation from 1901 to 2000. We used annual global atmospheric CO_2_ concentration data from ice-core records and atmospheric observations (Keeling and Whorf 2005), and soil texture data derived from the FAO soil datasets (Zobler 1986; FAO 1991). Ten climate change scenarios (2001–2100) were used, including four from the Intergovernmental Panel on Climate Change (IPCC) emission scenarios, HadCM3 A1FI, A2, B1, and B2 (Nakicenovic et al. 2000), and six (X901M, X902L, X903H, X904M, X905L, X906H) estimated by the MIT Integrated Global System Model (IGSM; Sokolov et al. 2005) for two emission scenarios used in the Synthesis and Assessment Product 2.1 of the U.S. Climate Change Science Program (Clarke et al. 2007).

The IPCC HadCM3 A1FI, A2, B1, and B2 scenarios are distinct emission scenarios that reflect the implementation of specific policies for controlling anthropogenic greenhouse gases in the future (Table 1). The six MIT IGSM climate scenarios were derived from ensemble simulations of potential climate responses to two emissions scenarios (reference and level 1 stabilization cases, Clarke et al. 2007; Webster et al. 2009) simulated by the MIT Emission Prediction and Policy Analysis (EPPA) model (Paltsev et al. 2005). Results from three simulations for each emission scenario were chosen from the ensemble simulations to provide climate inputs to LPJ-DGVM based on three sets of values for model parameters describing climate sensitivity, rate of heat uptake by the deep ocean, and the strength of aerosol forcing. One set represents median (M) values from the distribution of climate parameters obtained by Forest et al. (2008) and used in Sokolov et al. (2009). Simulation with this set of model parameters produces surface warming close to a median of the distribution obtained by Sokolov et al. (2009). The two other sets are the values of the parameters used in simulations that produce changes in surface air temperatures (SAT) close to the lower and upper bounds of the 90% range in climate response, or low (L) and high (H) climate response, respectively, obtained in the simulations for each emissions scenario (Sokolov et al. 2009). X901M, X902L, and X903H correspond to the reference emissions scenario, and represent the median, low, and high climate response, respectively (Table 1). Similarly, X904M, X905L, and X906H correspond to the level 1 stabilization scenario, and represent the median, low, and high climate response, respectively.

**Table 1:** Annual changing rate of air temperature (°C/year) and precipitation (mm/year) in the 10 climate scenarios used in this study, and the emission range (ppmv) from 2001 (*E*_2001_) to 2100 (*E*_2100_). The annual changing rates are determined as the slopes from a least squares linear regression using hundred-year time series from 2001 to 2100, over the region ranging from 45^o^N to 90^o^N. All estimates for air temperature and precipitation were statistically significant at *P* < 0.0001.

	Air temperature (°C/year)			Precipitation (mm/year)			Emission (ppmv)
Scenario	Estimate	95% CI	*R* ^2^	Estimate	95% CI	*R* ^2^	(*E*_2001_, *E*_2100_)
A1FI	0.136	(0.133, 0.140)	0.98	0.166	(0.161, 0.170)	0.98	(368.448, 925.531)
A2	0.106	(0.102, 0.109)	0.98	0.127	(0.123, 0.131)	0.98	(368.949, 818.891)
B1	0.056	(0.055, 0.057)	0.99	0.073	(0.071, 0.074)	0.99	(368.936, 531.371)
B2	0.069	(0.068, 0.069)	1.00*	0.086	(0.086, 0.087)	1.00*	(368.903, 603.702)
X901M	0.073	(0.071, 0.076)	0.97	0.117	(0.113, 0.122)	0.97	(370.957, 911.319)
X902L	0.057	(0.055, 0.058)	0.98	0.087	(0.084, 0.090)	0.97	(371.228, 947.953)
X903H	0.094	(0.090, 0.098)	0.96	0.148	(0.142, 0.155)	0.95	(371.281, 905.128)
X904M	0.019	(0.018, 0.020)	0.92	0.035	(0.032, 0.037)	0.87	(370.957, 476.506)
X905L	0.013	(0.012, 0.014)	0.85	0.023	(0.021, 0.026)	0.80	(371.228, 490.717)
X906H	0.027	(0.026, 0.028)	0.96	0.047	(0.044, 0.050)	0.93	(371.281, 472.149)

* Round to 1.00, not exactly equal to 1.

The annual changing rate of air temperature and precipitation for each climate scenario in the northern high latitudes are presented in Table 1, based on a simple linear regression for a time series of air temperature and precipitation, respectively. In particular, the mean annual air temperature between 2001 and 2100 over the domain significantly (*P* < 0.0001) increased throughout all scenarios. The largest increase (0.136°C/year) is from the A1FI climate scenario, and the smallest increase (0.013°C/year) is from the X905L climate scenario. Precipitation also significantly (*P* < 0.0001) increased across all climate scenarios, with the greatest increase (0.166 mm/year) occurring in the A1FI simulation and the smallest increase (0.023 mm/year) occurring in the X905L. The combination of these scenarios permits a robust analysis of the potential future response of vegetation dynamics to climate changes. In addition, the IGSM air temperature increases faster when the latitudes are higher. In contrast, the IPCC data have a much smoother and more consistent temporal increasing trend for annual temperature over the study domain.

As shown in Table 1, each scenario includes a projection of atmospheric CO_2_, starting from around 369 ppmv (IPCC) and 371 ppmv (IGSM) in 2001 and increasing to the magnitude of warming in year 2100, with the highest increase in the X902L scenario (948 ppmv) and the lowest increase in the X906H scenario (472 ppmv).

### MC simulations

The Latin Hypercube Sampling technique (LHS; Iman and Helton 1988) is based on MC simulations and uses a stratified sampling approach to efficiently estimate the output statistics (Van Griensven et al. 2006). With this approach, *n* samples were drawn for *k* random variables θ_1_, …, θ*_k_* over the feasible space described by their probability distributions. Following Tang and Zhuang (2009), three steps were performed to conduct the sampling. First, the distribution of each variable was subdivided into *n* strata with a probability of occurrence equal to 1/*n*. For uniform distributions, the variable range was subdivided into *n* equal intervals. Second, in each interval, one value was randomly selected with respect to the probability density in the interval. Third, the *n* values obtained for θ_1_ were randomly paired with those for θ_2_. Finally, these *n* pairs were further randomly paired with the *n* values of θ_3_ to form *n* triplets, and so forth, until *n* nonoverlapping realizations were created.

In this study, we used the LHS method to generate 1,000 sets of parameter samples for the LPJ MC simulations with each of the 10 climate datasets. Ranges of parameter values were assigned based on either literature review or estimates (Table 2). We neglected the correlations among the parameters when performing the sampling and each parameter was assumed to follow a uniform distribution. This assumption could have led to an overestimation of uncertainty and reduce the confidence in model results (Helton and Davis 2000; Zaehle et al. 2005). Future efforts could apply the inverse method to introduce parameter correlations and further constrain the model uncertainties (Kaminski et al. 2002; Rayner et al. 2005).

**Table 2:** Key Lund-Potsdam-Jena Dynamic Global Vegetation Model (LPJ-DGVM) parameters involved in this study.

Parameter	Standard	Range	Description	Reference
*k*_beer_	0.5	[0.4, 0.7]	Light extinction coefficient	Larcher (2003)
θ	0.7	[0.2, 0.996]	Photosynthesis colimitation shape parameter	Collatz et al. (1990), Leverenz 1988)
*k*_mort1_	0.05	[0.01, 0.01]	Asymptotic maximum mortality rate (yr^−1^);	Null
*k*_mort2_	0.5	[0.2, 0.5]	Growth efficiency mortality scalar	Null
α_a_	0.5	[0.3, 0.7]	Photosynthesis scaling parameter (leaf to canopy)	Haxeltine and Prentice (1996)
α_C3_	0.08	[0.02, 0.125]	Intrinsic quantum efficiency of CO_2_ uptake in C3 plants	Farquhar et al. (1980), Hallgren and Pitman (2000)
*k*_allom1_	100	[75, 125]	Crown area =*k*_allom1_× height *^krp^*	Null
*k*_allom3_	0.67	[0.5, 0.8]	Height =*k*_allom2_× diameter *^k^*^allom3^	Huang et al. (1992)
*k*_rp_	1.6	[1.33, 1.6]	Crown area =*k*_allom1_× height *^krp^*	Enquist and Niklas (2002), Whitehead et al. (1994)
*k*_la:sa_	4000	[2000, 8000]	Leaf to sapwood area ratio	Waring et al. (1982)
*CA*_max_	15.0	[7.5, 30.0]	Maximum woody PFT crown area	Null
*r*_growth_	0.25	[0.15, 0.4]	Growth respiration per unit Net Primary Production (NPP)	Sprugel et al. (1995)
*est*_max_	0.12	[0.05, 0.3]	Maximum sapling establishment rate [m^−2^yr^−1^]	Null

For each LHS set, the LPJ simulation was started from “bare ground,” where no plant biomass existed. Then following Sitch et al. (2005), a “spin-up” simulation of thousand years was conducted using a cyclic replication of CRU monthly climatology from 1901 to 1930 to reach approximate equilibrium with respect to carbon pools and preindustrial stable vegetation cover. Then, the transient historical climate (CRU, 1901–2000) and projected climate change scenarios (e.g., IPCC A2, 2001–2100) were used to drive the model. We ran the simulations for all 10 climate change scenarios and compared the FPC output of each PFT. The temporal trends of all PFT area changes were presented for the current century. We also compared results from the MC ensemble simulations with those from control simulations (with default parameter values from previous literature, Sitch et al. 2003), both driven by 10 different climate scenarios.

### Sensitivity and uncertainty analysis

We used the PRCC (Marino et al. 2008; Saltelli 2002), a sensitivity index based on linear (respectively monotonic) assumptions, to identify the relative importance of the contribution from a particular parameter to the simulation uncertainty. We briefly describe how we calculated the PRCC below.

A correlation coefficient (CC) between input *x_j_* and output *y* was calculated as

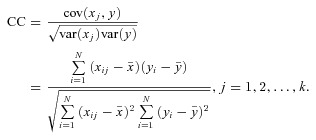
1
where cov(*x_j_*,y) represents the covariance between *x_j_* and *y*, while var(*x_j_*) and var(*y*) are the variances of *x_j_* and *y*, respectively. The respective sample means of *x* and *y* are 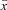 and 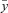.

Partial correlation characterizes the linear relationship between input *x_j_* and output *y* after the linear effects on *y* of the remaining inputs are discounted. The partial correlation coefficient (PCC) between *x_j_* and *y* represents the CC between two residuals, 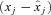 and 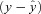, where 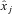 and 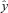 are the estimated values of the linear regression

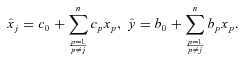
2
To measure the potential nonlinear but monotonic relationships between *x_j_* and *y*, we calculated the PRCC, which is a robust measure as long as little to no correlation exists between inputs (Marino et al. 2008). It represents a partial correlation on rank-transformed data (x*_j_* and *y* are first rank transformed and then the linear regression models in eq. 2 are built). A higher positive PRCC indicates that the parameter has a greater positive control on the response variable of interest, while a higher (absolute value) negative PRCC indicates a greater negative control. In contrast, a PRCC value close to 0 indicates a poor effect on the response variable of interest. The free software package R version 2.11.1 and the “sensitivity” package for R were used for the calculation of PRCCs (Pujol et al. 2008; RDC–Team 2010).

In this study, of the total 36 parameters, we selected 13 key parameters (Table 2), which are demonstrated as the most important ones in the determination of FPC in Zaehle et al. (2005). A detailed definition of each parameter can be found in Zaehle et al. (2005). Parameter importance of these 13 parameters was determined for all 0.5 × 0.5 grid cells (totally 25,063 cells) in the region. We used the mean hundred-year FPC values (from 2001 to 2100) to analyze the parameter importance for each PFT. Since LPJ uses the same parameters to describe a specific PFT in different regions experiencing distinct climate conditions, it is necessary to test the sensitivity of parameter importance to climate inputs. Based on 10,000 MC simulations, we compared the parameter importance among the ten different climate change scenarios. To investigate how vegetation dynamics are sensitive to climate change, we examined the spatial pattern of climate change and how it is associated with the change of each PFT in the region north of 45^o^N.

Under each climate change scenario, we produced the mean, variance, and the 90% confidence interval (CI) of the projected vegetation coverage for a specific PFT, based on the 1000 MC simulations. The variance and the 90% CI represent the parameter-based uncertainty in modeled vegetation distribution. We also calculated the mean and variance associated with the climate-induced uncertainties under each climate scenario.

## Results

### Parameter importance

Parameters controlling the competitive strength of a specific PFT and the interspecies competition balance determine the variation of FPC. Unlike Zaehle et al. (2005), we included the relative parameter importance in determining the FPC of each individual PFT, not only for the dominant, subdominant, and herbaceous PFTs in grid cells.

Based on the absolute value of the PRCC, we ranked the importance of the 13 selected parameters (Table 3). The most influential parameters in determining vegetation distribution include α_C3_, *k*_rp_, and *k*_beer_. Parameters of secondary importance include *est*_max_, *k*_la:sa_, α_a_, and *k*_allom1_. The remaining six parameters are relatively less important than the parameters in the first two groups, and are organized in the third importance group. Table 3 shows that the ranking of relative parameter importance could vary among different PFTs; the general order, however, is similar.

**Table 3:** Partial Rank Correlation Coefficients (PRCCs) for the projected area of a specific plant functional type and each input parameter. These PRCCs are calculated using the average foliage projective cover (FPC) outputs across all climate scenarios.

	TNE		TBE		TBS		BNE		BSW		CPG	
Rank	Parameter	PRCC	Parameter	PRCC	Parameter	PRCC	Parameter	PRCC	Parameter	PRCC	Parameter	PRCC
1	α_C3_	0.678	α_C3_	0.784	α_C3_	0.697	*k*_beer_	−0.781	α_C3_	0.778	*k*_beer_	0.899
2	*k*_beer_	–0.610	*k*_rp_	–0.655	*k*_beer_	–0.614	α_C3_	0.373	*k*_beer_	–0.657	α_C3_	–0.728
3	*k*_rp_	–0.528	α_a_	0.512	*k*_rp_	–0.599	*est*_max_	0.294	*k*_rp_	–0.533	*k*_rp_	0.655
4	*k*_la:sa_	–0.467	*k*_beer_	0.511	α_a_	0.407	*k*_la:sa_	–0.239	α_a_	0.502	*est*_max_	–0.506
5	α_a_	0.383	*k*_la:sa_	–0.363	*est*_max_	0.383	*k*_rp_	–0.206	*k*_la:sa_	–0.298	*k*_allom1_	–0.443
6	*k*_allom1_	0.296	*k*_allom1_	0.362	*k*_allom1_	0.351	*k*_allom1_	0.181	*r*_growth_	–0.291	*k*_la:sa_	0.438
7	*est*_max_	0.278	*r*_growth_	–0.291	*r*_growth_	–0.317	α_a_	0.171	*k*_allom1_	0.266	α_a_	–0.418
8	θ	0.096	*est*_max_	0.269	*k*_allom3_	0.301	*k*_mort1_	–0.129	*k*_mort1_	–0.255	*k*_mort1_	0.28
9	*k*_allom3_	0.085	θ	0.2	*k*_la:sa_	–0.212	*CA*_max_	0.098	*est*_max_	0.251	*k*_allom3_	–0.243
10	*CA*_max_	–0.085	*k*_allom3_	0.173	θ	0.189	*k*_allom3_	0.038	*k*_allom3_	0.196	θ	–0.175
11	*r*_growth_	–0.034	*CA*_max_	–0.13	*k*_mort1_	–0.138	*r*_growth_	–0.033	θ	0.165	*r*_growth_	0.144
12	*k*_mort2_	–0.014	*k*_mort1_	0.114	*CA*_max_	–0.105	θ	–0.012	*CA*_max_	–0.075	*k*_mort2_	–0.141
13	*k*_mort1_	–0.006	*k*_mort2_	–0.111	*k*_mort2_	0.013	*k*_mort2_	–0.002	*k*_mort2_	0.063	*CA*_max_	0.007

TNE, temperate needleleaved evergreen tree; TBE, temperate broadleaved evergreen tree; TBS, temperate broadleaved summergreen tree; BNE, boreal needleleaved evergreen tree; BSW, boreal summergreen woody tree; CPG, C3 perennial grass.

**Figure 1:**
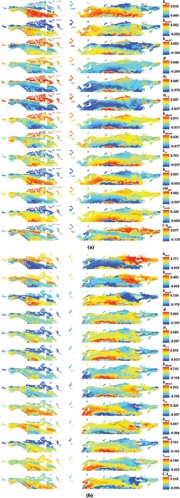
Parameter importance measured as Partial Rank Correlation Coefficient (PRCC) of 13 parameters for the foliage projective cover (FPC) of (a) C3 perennial grass, and (b) boreal needleleaved evergreen trees based on 10,000 Lund-Potsdam-Jena Dynamic Global Vegetation Model (LPJ-DGVM) ensemble simulations under 10 different climate change scenarios in the northern high latitudes. *Note.* For a description of each parameter refer to Table 2. If a specific plant functional type (PFT) never appears in a grid cell, the grid cell is then not considered when mapping.

For a certain PFT, the influence of each parameter on the corresponding FPC output exhibits a pattern of distinct spatial characteristics. For example, *k*_beer_ has a relatively low influence on the CPG coverage in the high Arctic region (Fig. 1a), and a high influence on CPG coverage in the southern region (e.g., Canadian boreal forest). This implies that the increased available radiation as a result of reduced PAR within the canopy has less effect on the FPC of CPG in the very north region. Among different PFTs, the spatial variation in the influence of parameters exhibits both similarities and differences. For instance, unlike CPG, the BNE coverage in the Siberia zone is strongly controlled by *k*_beer_ (Fig. 1b). This implies that in the Siberia zone, the elevated available radiation due to reduced canopy PAR exerts less of an effect on the FPC of CPG than on that of BNE trees. Other parameters also exhibit quite different regional characteristics in the determination of the FPC output of each PFT.

**Figure 2:**
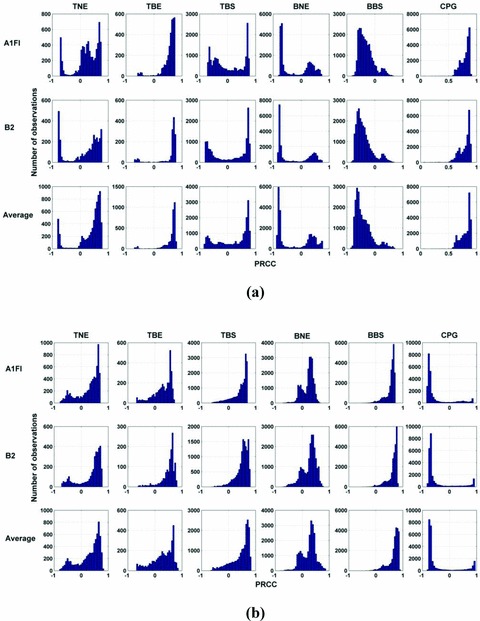
Histogram of the PRCCs of (a) *k*_beer_ and (b) αC3 for six PFTs in all grid cells in which a specific PFT survives from 2001 to 2100. From top to bottom, results are determined under A1FI, B2, and averaged climate scenarios. From left to right, results are for temperate needleleaved evergreen (TNE) woody, temperate broadleaved evergreen (TBE) woody, temperate broadleaved summergreen (TBS) woody, boreal needleleaved evergreen (BNE) woody, boreal summergreen woody (BSW), and C3 perennial, respectively.

Our study shows that climate input potentially affects the expansion of influence of all 13 parameters. Based on PRCCs of all 25,063 grid cells, Figure 2a shows that histograms of the PRCC values of *k*_beer_ could be different among different climates (e.g., A1FI and A2). For example, under the A1FI climate scenario, more grid cells were positively affected by *k*_beer_ for the TNE coverage, compared with the A2 scenario. A major reason is that under the A1FI condition, the TNE trees could survive in more grid cells, therefore contributing to the larger extent of *k*_beer_ influence. Furthermore, distributions of PRCCs for *k*_beer_ influencing the TNE coverage are different between the A1FI and A2 conditions. Similar to *k*_beer_, the expansion of α_C3_ could also be affected by climates. As shown in Figure 2b, less grid cells were influenced by α_C3_ for the TBE coverage under the A2 climates, than those under the A1FI climate. For different PFTs, the distribution of PRCCs for a certain parameter is quite different under the same climate scenario. Additionally, among different parameters, the distributions of PRCCs are also very different for the coverage of a specific PFT under the same climate.

**Figure 3:**
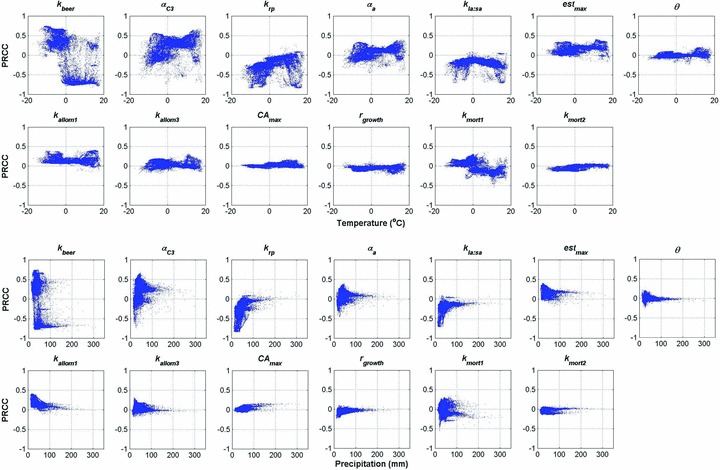
Scatter plot of PRCCs versus mean air temperature (°C) or mean precipitation (mm) for all grid cells (out of total 25,063 grids), which have valid PRCC values for BNE woody under the A1FI scenario.

**Figure 4:**
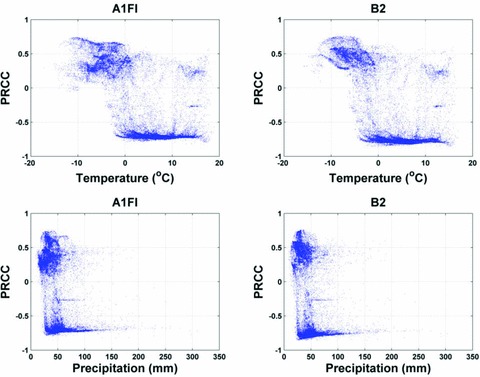
Scatter plot of PRCCs for *k*_beer_ versus mean air temperature (°C) or mean precipitation (mm) for all grid cells (out of total 25,063 grids), which have valid PRCC values for BNE woody under the A1FI and B2 scenarios.

To examine the potential effect of climate on parameter importance, we investigated the relationship between PRCCs and a specific climate input (i.e., air temperature or precipitation). For the total 25,063 grid cells, Figure 3 exhibits the PRCC values (for BNE trees) versus the mean annual air temperature or precipitation from the A1FI scenario. We found that some parameters (e.g., *k*_beer_ and *k*_rp_) are sensitive to temperature when determining the BNE coverage. In particular, *k*_beer_ generally exerts a positive effect when temperature is below zero and a negative effect when temperature is above zero (Fig. 3). The relative importance (measured using PRCC) of *k*_rp_ has a general decreasing trend as temperature increases. Parameters such as α_C3_, *k*_rp_, and α_a_ seem to be vulnerable to precipitation as shown in Figure 3. These parameters exhibit a strong influence (relatively high absolute value for PRCC) in grid cells with low precipitation, and moderate influence in grid cells with high precipitation. However, parameters (e.g., *k*_mort2_) exhibiting a flat scatter plot had low sensitivity to air temperature or precipitation changes. Under a different climate (e.g., B2), the scatter plot could be slightly, but not significantly changed, and the pattern of the plot will remain almost the same in all cases. For instance, the responses of *k*_beer_ to air temperature and precipitation are similar when the B2 scenario is used, compared with the responses when the A1FI scenario is used (Fig. 4).

### Parameter-induced uncertainties in modeling coverage for each PFT

**Figure 5:**
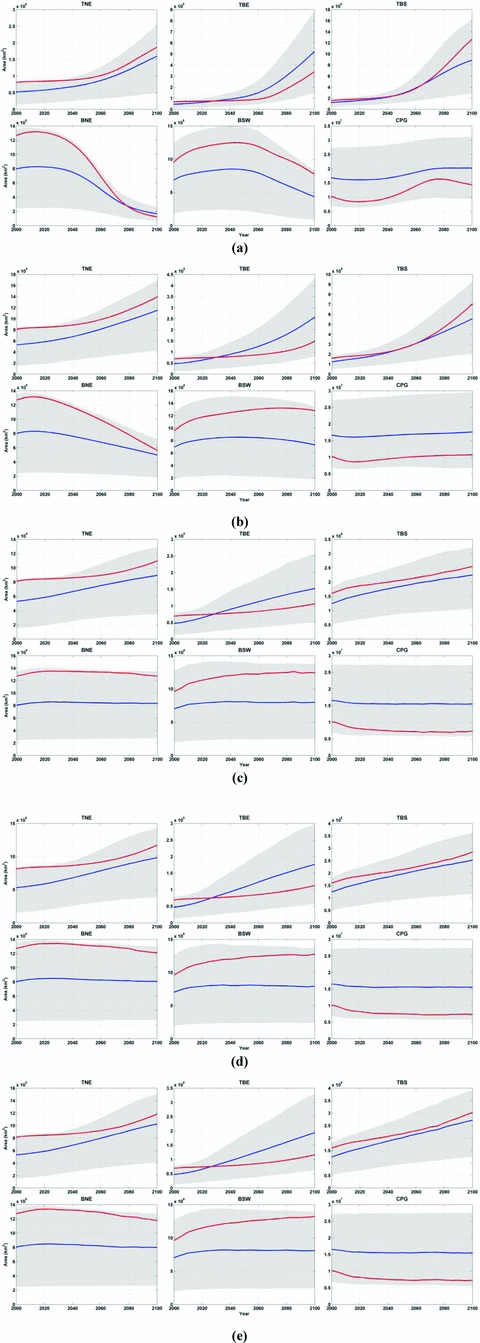
The averaged coverage (km^2^) of each PFT from the Monte Carlo (MC) ensemble simulations ( blue lines) with their uncertainty ranges (shaded regions), and results from control simulations (red lines) using default parameter values from Sitch et al. (2003) from 2001 to 2100, under (a) A1FI, (b) B2, (c) X905L, (d) X904M, and (e) X906H scenarios. The upper and lower bounds of the uncertainty range are determined by the 90% and 10% quantiles based on the MC ensemble simulations. TNE, temperate needleleaved evergreen; TBE, temperate broadleaved evergreen; TBS, temperate broadleaved summergreen; BNE, boreal needleleaved evergreen; BSW, boreal summergreen woody; CPG, C3 perennial grass.

Under a certain climate, the uncertainty in projecting vegetation coverage is attributed to the uncertainty in parameter values or their combinations, especially the most important ones. Figure 5 shows considerable parameter-induced uncertainty in each PFT's coverage under different climates. The upper and lower bounds of the uncertainty range are determined by the 90% and 10% quantiles, respectively. With a certain combination of parameters, different climate inputs resulted in diverged PFT coverage since the changing rate and magnitude of air temperature, precipitation, and CO_2_ concentration are quite different among different climate scenarios (Table 1). Because the range of parameter-induced uncertainty is determined by the combination of extreme parameter values, a slight divergence in the uncertainty range is found among different climates (Fig. 5). For example, under the reference emission scenario, the intense climate (i.e., X903H) results in the largest uncertainty in the projected TNE, TBE, and TBS coverage. X901M and X902L result in the moderate and lowest variation of the vegetation coverage, respectively. However, variations in BNE, BSW, and CPG areas exhibit no clear differences among these three different climate scenarios. Under the stabilized emission scenario, the uncertainty ranges for all six PFTs are very similar through the high (X906H), moderate (X904M), and low (X905L) climate conditions.

Compared with control outputs, the ensemble mean outputs always underestimated the TNE, BNE, and BSW coverage, and overestimated the CPG coverage (Fig. 5). Nevertheless, the mean ensemble outputs for TBE and TBS coverage are both comparable to those from control simulations. This suggests that current LPJ parameterization for TBE and TBS plants leads to an “average” output as a consequence of the large amount of combinations of parameter values.

From Figure 5, we found that the parameter-based uncertainty in TNE coverage is approximately equivalent to 60%∼ 70% of the mean ensemble output. A large part of this uncertainty is due to uncertainties in α_C3_ (PRCC = 0.678), *k*_beer_ (PRCC =–0.610), *k*_rp_ (PRCC =–0.528), and *k*_la:sa_ (PRCC =–0.467; Table 3). TNE coverage seems to be most likely underestimated in our ensemble simulations, compared with the control output. For TBE trees, besides the above four parameters, the variation of *k*_rp_ (PRCC =–0.655) also contributes much to the uncertainty of TBE coverage. The uncertainty range of the TBE area is close to 80% of the mean output. Variations in α_C3_ (PRCC = 0.697), *k*_beer_ (PRCC =–0.614), and *k*_rp_ (PRCC =–0.599) significantly influence the uncertainty of TBS coverage, of which the projected mean coverage is in agreement with that of the control simulation. The variation due to parameter-based uncertainty exists in a range equivalent to 70% of the mean coverage. For BNE plants, *k*_beer_ (PRCC =–0.781) is the most important parameter in determining the projected BNE coverage. For BSW and CPG, α_C3_, *k*_beer_, and *k*_rp_ mostly control the uncertainty ranges. Variations due to parameter-based uncertainty approximately equal to 80% of the mean values.

### Emission- and climate-induced uncertainty

**Figure 6:**
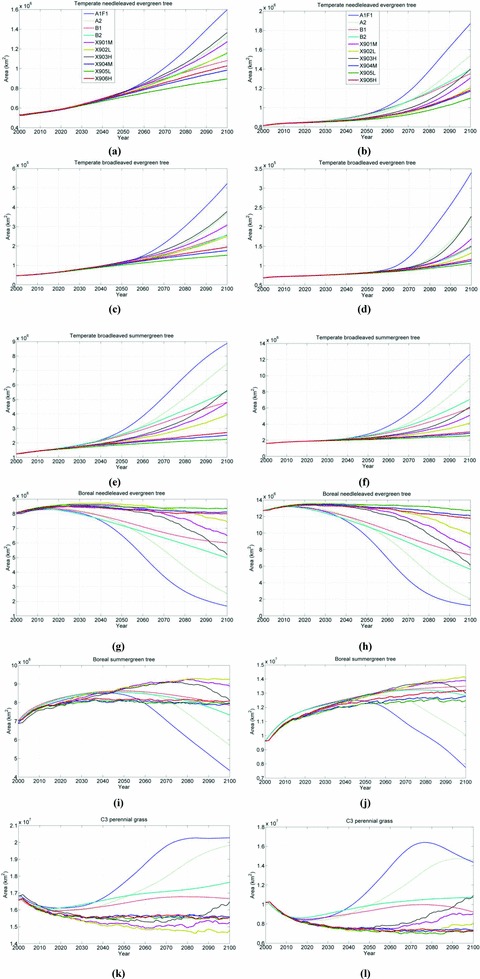
Temporal change of simulated mean areas (km^2^, left) and results from the control simulations (right) of six PFTs under 10 climate change scenarios, from 2001 to 2100. X901M, X902L, and X903H correspond to the reference emission scenario; X904M, X905L, and X906H correspond to the stabilization emission scenario.

Simulations driven by four IPCC emission scenarios (i.e., A1FI, A2, B1, and B2) provide an opportunity to explore the influence of emission scenarios on vegetation distribution. In Figure 6, differences in coverage of a certain PFT among different scenarios could be related to variability in emission scenarios. In addition, the intense, moderate, and weak scenarios correspond, respectively, to the largest, moderate, and lowest uncertainties in projecting TNE, TBE, and TBS coverage (Fig. 5). However, for BNE, BSW, and CPG, uncertainty ranges are similar among these four emission scenarios.

The MIT IGSM simulations represent the climate uncertainty that might result from a single emissions scenario (e.g., “reference” and “stabilization”). Thus, unlike the simulations using IPCC emissions scenarios, the results from the MIT IGSM simulations can be used to account for both climate uncertainty and the LPJ parameter uncertainty on the projected area of PFTs. Here, we used the differences between results from the high climate response (e.g., X903H and X906H) or the low climate response (e.g., X902L and X905L), and those from median climate response (e.g., X901M and X904M) to determine the climate-induced uncertainty range. Under a certain emission scenario (e.g., reference emission), differences in coverage of a certain PFT among different climates (X901M, X902L, X903H), as shown in Figure 6, could be related to climate uncertainties. Compared with the parameter-induced uncertainty (Fig. 5), the climate-induced uncertainty is much smaller, but still significant. Comparing the results from two different emission scenarios (reference vs. stabilization) as shown in Figure 6, we found that the difference in emission scenarios contributes much to the variation of each PFT coverage. The emission-induced uncertainty is even larger than the climate-induced uncertainty, but still smaller than the parameter-induced uncertainty.

To explore the various vegetation responses to climate, we also examined the relationship between FPC outputs and a specific climate factor (i.e., air temperature and precipitation). Using FPC outputs (averaged from 2001 to 2100) from all grid cells (which total 25,063), we performed a simple linear regression between FPC and mean air temperature, mean precipitation, slope of air temperature, and slope of precipitation, separately. An obvious correlation exists between the TBS coverage with the mean air temperature (*R*^2^ > 0.55 under all climates). However, the slope of air temperature shows little correlation with FPC outputs. Although the mean precipitation shows no strong correlation on FPCs of all PFTs, a modest linear relationship exists between slopes of precipitation for four IPCC HadCM3 climates as well as for TBS coverage (*R*^2^ > 0.3).

### Projection of future vegetation distribution

#### Temporal changes

Changes of vegetation distribution in the current century are projected to be different under different climate scenarios (Fig. 6). Generally, higher emission scenarios (i.e., reference emission) result in a higher changing rate of vegetation redistribution, compared with those under the lower emission scenarios (i.e., stabilized emission scenario).

In particular, for TNE trees, both ensemble simulations (Fig. 6a) and the control simulation (Fig. 6b) predicted a significant area increase across all climates, with the highest change occurring under the A1FI climate (an approximately 1.1 × 10^6^ km^2^ increase) and the lowest change occurring in the X905L climate (an approximately 0.2 × 10^6^ km^2^ increase). A similar trend was projected for TBE plants. The projected TBS area exhibited a clear increasing trend under seven climate scenarios, which included all except the three climates under the stabilization emission scenario (Fig. 6e and 6f). Unlike the TBS trees, the BNE coverage had an obvious shrinking trend under most climates, except under the stabilization emission scenario (Fig. 6g and 6h). The largest decrease happened under A1FI (approximately 90% decreases) and the lowest occurred under X902L (less than 10% decreases). Driven by four IPCC climates, ensemble simulations produced first an increasing followed by a decreasing trend in BSW coverage with the turning point located in the mid-century (Fig. 6i). A similar trend was found under the reference emission scenario from MIT IGSM; however, the decreasing amplitudes were much lower and the turning point occurred much later (approximately 2080). In contrast, under the stabilization emission scenario, there were almost no changes after the BSW area achieved its peak in 2030. Finally, only two climates (A1FI and B2) lead to a net decrease in BSW area in both types of simulations (Fig. 6i and 6j). For CPG plants, two climates (A1FI and A2) lead to a substantial coverage increase at the end of the 21st century, while the other climates contributed to a slight increase or a net decrease in CPG coverage (Fig. 6k and 6l). It should be noted that in the control simulation under A1FI, CPG coverage shrank significantly after reaching its peak in the year 2075 (Fig. 6l).

#### Spatial changes

Among different climates, the changing rates of vegetation distribution are quite different and more pronounced under strong warming scenarios (e.g., A1FI, Fig. 6). For the Arctic region, we projected a northward grass expansion associated with warming temperatures. As the warming conditions are projected to continue into the 21st century, we predicted a continuous increase of greenness in this region. We found that different climates result in different extents of greenness in the region. For example, the most extreme warming climate, A1FI, is responsible for the intense tree line expansion, while the lowest warming climate, X905L, corresponds to the weakest tree line migration.

**Figure 7:**
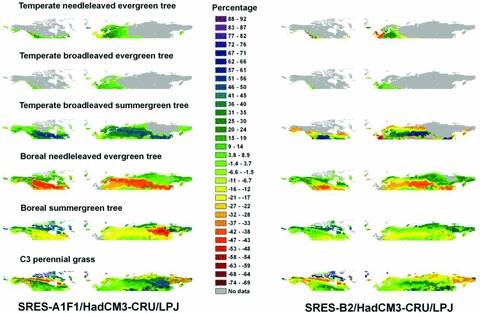
Changes in vegetation distribution of six PFTs between 2000 and 2100. Simulated changes in FPC were calculated under a strong (HadCM3-A1FI) and a moderate climate change scenario (HadCM3-B2).

In North America and Eurasia, we projected a clear northward expansion of BSW plants into the low Arctic region (Fig. 7). However, it is worth noting that the increased temperature strongly influences the competitive advantage between BSW and CPG plants. For instance, under A1FI, BSW plants would replace a large area of CPG due to warming temperatures in the region during the first half of the 21st century. However, in the second half century, the continuous increasing temperature completely reverses the competitive advantage between BSW and CPG. Consequently the CPG plants could redominate large areas once covered by BSW plants. Meanwhile, increased temperature enhances evapotranspiration, thereby resulting in “drier” conditions that strengthen the competition of CPG, even if the region receives more precipitation.

A unanimous decline in BNE coverage is predicted in western Eurasia and North America, due to the replacement of CPG plants. However, the extent of the decline is largely controlled by warming temperatures. In particular, the intense warming scenario (e.g., A1FI) is always responsible for the most intense response (Fig. 7). In contrast, under X905L, BNE trees are always the dominant vegetation in boreal forest ecosystems, despite the small northward migration of TBS trees in Western Europe. During the first half of the 21st century, we projected a strong northward CPG expansion from the drying and less warming region (45^o^N–55^o^N) in Eurasia. However, the continuous warming temperatures could cause increased dryness, which would further favor the domination of TBS trees in the grass-migrated boreal forest regions in the second half of the 21st century. In North America and Eurasia, we projected a significant northward expansion of TBS trees. Our findings are consistent with Wolf et al. (2008), which predicted a more deciduous world in terms of land vegetation in the 21st century. The TNE plant was always, in our simulations, the dominant PFT in western Eurasia, and in the small area along western Canada. TBE plants appear in the same regions as TNE trees, and also have a temporal increasing trend, but do not spread into other regions.

## Discussion

### Uncertainty of vegetation distribution

In this study, the parameter-induced uncertainty contributes most to the total uncertainty in projecting vegetation distributions (Fig. 5). Since the uncertainty range is determined by the consequences of parameter combinations with extreme parameter values, a further constrained parameter space could potentially reduce the uncertainty range. One widely used method to constrain the model uncertainty is “the inverse method,” which is able to introduce parameter correlations (Kaminski et al. 2002; Rayner et al. 2005). Furthermore, some parameters could be obtained through field experiments (e.g., *CA*_max_).

Because each parameter either governs a biogeophysical or biogeochemical process, or represents an ecological constant, detailed information for a spatial analysis of parameter importance (Fig. 1) provides useful information for the assessment of regional environmental effects on vegetation coverage. However, we acknowledge that several important processes are not explicitly parameterized or described in the current model. For example, the fire return interval (Jiang et al. 2009; Jiang and Zhuang 2011) and successional pathways that the vegetation follows after a fire event are important, but these types of dynamics and their associated parameters are not fully modeled here. This is also true for permafrost dynamics and the nitrogen cycle. Under a warming climate, permafrost exerts significant effect on vegetation change through increased thawing of the active layer depth (Euskirchen et al. 2006; Tchebakova et al. 2009; Zhuang et al. 2003). Consequently, this would result in a complex variety of vegetation responses. On the one hand, a northward tree line expansion would follow permafrost retreat. On the other hand, thermokarst development in the boreal forest region could remove the ice-rich substrate, which is the physical foundation of the forest, thereby destroying extensive spruce and birch forests and leading to a conversion to wet sedge meadows (Osterkamp et al. 2000). In addition, nitrogen dynamics considerably impact plant growth and productivity by limiting nitrogen availability for carbon sequestration (LeBauer and Treseder 2008). In this study, the effect of CO_2_ fertilization on the northward greenness expansion could be somewhat overestimated because uncoupled nitrogen dynamics could realistically limit the rate of plant shift. In addition to parameters, the climate variability also contributes to the uncertainty of projected vegetation coverage (Fig. 5). All six PFTs seem to be sensitive to climate variability; however, different PFTs present distinct behaviors in response to climate variability. These differences are associated with the different bioclimatic limits (e.g., the lower and upper limit of temperature optimum for photosynthesis) used for each PFT in the model.

The regional characteristics of vegetation redistribution are indeed determined by the regional climate. However, other regional factors, such as permafrost dynamics (Wania et al. 2009a, b), human influence (Neilson et al. 2005; Araújo and Rahbek 2006; Araújo and New 2007), and topographic constraints (Rupp et al. 2001) could also be key determinants in the control of vegetation dynamics in the northern high latitudes. For example, human land use exerts considerable effects on landscape change, and through fire suppression, human activities strongly influence the wildfire regime, which is a major disturbance in boreal forest ecosystems (Kasischke et al. 2002; Rupp et al. 2002; Kasischke and Turetsky 2006; Flannigan et al. 2009). In addition, the topographic barrier is always an important limitation to tree line advance, especially in mountain areas.

The emission-induced uncertainty was examined by stratifying comparisons of FPC outputs using the climates of X901M, X902L, and X903H separately from results using the climates of X904M, X905L, and X906H. Our results imply that the CO_2_ fertilization effect on plants is substantial, especially in the high-emission scenarios (e.g., reference emission from MIT IGSM), and thus may make a large contribution to vegetation change, in addition to the contribution it makes to direct temperature and water effects. This is consistent with other studies (e.g., Bachelet et al. 2003) that have shown that the rate of vegetation change would be affected by the extent of the CO_2_ fertilization effect. In this study, between the reference and stabilization emission scenarios, the magnitudes of the uncertainty range are quite different (Fig. 6). In particular, under the reference emission scenario, uncertainties in FPC outputs are generally larger than those under the stabilized emission scenario. An analysis of emission-induced uncertainty for future vegetation distribution provides useful information for policy makers and ecosystem managers to better understand the effects of CO_2_ fertilization on vegetation dynamics and the potential northward shift of greenness in the northern high latitudes.

### Future vegetation distribution and its consequences

Compared with control outputs, we projected a northern hemisphere with less TNE, BNE, and BSW trees, and more CPG plants based on the ensemble simulations (Fig. 5). This implies that the environmental changes (e.g., changes of biogeophysical or biogeochemical processes) are more likely to contribute to increases in TNE, BNE, and BSW coverage, and to a decrease in CPG coverage. For TBS trees, it suggests that the control simulation with its current parameterization can somewhat represent the “average” output from a large number of simulations with different combinations of parameter values.

In our simulations, climate input (e.g., air temperature) plays an important role in determining vegetation redistribution; this has also been demonstrated in other recent studies (Kharuk et al. 2009; Shuman and Shugart 2009; Giesecke et al. 2010; Lantz et al. 2010; Wright and Fridley 2010). For example, the continuous increased summer temperature in the Arctic region potentially achieves the lowest requirement for CPG to survive, therefore stimulating northward CPG expansion in the region. In the Siberia region, the warmer and wetter climate first stimulates the BSW expansion, and then the highly increased temperature potentially results in a much drier condition that exerts strong water stress for BSW trees and consequently leads to their recession. The projected BNE decline is associated with the increasing heat stress (leading to possible high peak tissue temperature) on BNE trees (Lucht et al. 2006) throughout all climate scenarios. Compared with previous studies (e.g., Chapin and Starfield 1997; Rupp et al. 2001; Neilson et al. 2005), which suggest that the northward treeline migration into the western Arctic region would likely take centuries to occur, our results show that this time scale could be much shorter. However, since many (species-specific) processes, habitat and landscape fragmentation, invasions, plasticities, and so on, are not included in the model, it may well be biased in terms of the speed of vegetation distributional and compositional changes.

Consistent with previous studies (e.g., Euskirchen et al. 2009), our results indicated a complexity of regional vegetation responses to future climate change in the northern high latitudes (Fig. 7). However, a unanimous northward treeline migration due to anomalous warming conditions was projected in the Arctic region across all climates, which is consistent with other studies (Lloyd and Fastie 2002; Bachelet et al. 2005; Scholze et al. 2006; McGuire et al. 2007; Wolf et al. 2008). The predicted expansion of greenness in the Arctic region (Fig. 7) could dramatically change the regional features, which were originally characterized by low air and soil temperatures, permafrost, a short growing season, and limited vegetation productivity (Stow et al. 2004). In particular, the projected northward tree line expansion could decrease the surface albedo (Thompson et al. 2004) and lead to a substantial positive feedback on atmospheric heating in summer (Chapin III et al. 2005; Euskirchen et al. 2009). Subsequent environmental responses, such as a decrease in snow cover duration and extent (Euskirchen et al. 2006, 2007; Stone et al. 2002), the exacerbation of permafrost degradation (Jorgenson et al. 2001), and the lengthening of the growing season (Smith et al. 2004) could further facilitate the surviving of trees and grasses.

Since the structure and composition of plant communities have a fundamental effect on both regional and global climate systems as they modify surface albedo, carbon fluxes, and the water cycle (Harding et al. 2002; Callaghan et al. 2004; ACIA 2005), the detailed analysis of parameter importance and regional vegetation changes presented in this study provides useful information for further analysis of ecological responses (e.g., water and carbon cycle) to potential climate changes and future ecosystem resource management schemes.

## Conclusions

This study quantitatively investigated the parameter and climate-induced uncertainties in simulating vegetation distribution in the northern high latitudes using LPJ. Parameter-induced uncertainties contribute the largest amount of uncertainty in modeling vegetation distribution, while emission- and climate-induced uncertainties are also significant. The relative importance of different parameters varies temporally and spatially in the region, and is influenced by climate inputs. Parameters controlling plant C uptake and light-use efficiency have the predominant influence in determining the vegetation coverage of both woody and herbaceous plant function types. Although the uncertainty is significantly large, we projected a unanimous northward greenness in the Arctic region due to anomalous warming. Temporally, boreal needleleaved evergreen plants are projected to decline considerably, and a large portion of C3 perennial grass will disappear by the end of the century. In contrast, the area of temperate trees would increase considerably. This study provides useful information in the exploration of the influence of environmental changes on vegetation dynamics, and the different vegetation responses to different emission polices.
